# Fostering global data sharing: highlighting the recommendations of the Research Data Alliance COVID-19 working group

**DOI:** 10.12688/wellcomeopenres.16378.2

**Published:** 2021-05-26

**Authors:** Claire C. Austin, Alexander Bernier, Louise Bezuidenhout, Juan Bicarregui, Timea Biro, Anne Cambon-Thomsen, Stephanie Russo Carroll, Zoe Cournia, Piotr Wojciech Dabrowski, Gayo Diallo, Thomas Duflot, Leyla Garcia, Sandra Gesing, Alejandra Gonzalez-Beltran, Anupama Gururaj, Natalie Harrower, Dawei Lin, Claudia Medeiros, Eva Méndez, Natalie Meyers, Daniel Mietchen, Rajini Nagrani, Gustav Nilsonne, Simon Parker, Brian Pickering, Amy Pienta, Panayiota Polydoratou, Fotis Psomopoulos, Stephanie Rennes, Robyn Rowe, Susanna-Assunta Sansone, Hugh Shanahan, Lina Sitz, Joanne Stocks, Marcos Roberto Tovani-Palone, Mary Uhlmansiek, Research Data Alliance

**Affiliations:** 1 Environment and Climate Change Canada, 351 boul. St-Joseph, Gatineau, Quebec, K1A 0H3, Canada; 2 Centre of Genomics and Policy, McGill University, 740, avenue Dr. Penfield, suite 5200, Montreal, Quebec, Canada; 3 Institute for Science, Innovation and Society, University of Oxford, 64 Banbury Road, Oxford, OX2 6PN, UK; 4 UKRI-STFC Rutherford Appleton Laboratory, Harwell Campus, Didcot, OX11 0QX, UK; 5 Digital Repository of Ireland, Royal Irish Academy, 19 Dawson St, Dublin 2, D02 HH58, Ireland; 6 CNRS, Inserm and University Toulouse III Paul Sabatier, Toulouse, France; 7 Native Nations Institute at the Udall Center for Studies in Public Policy and the College of Public Health, University of Arizona, 803 E First ST, Tucson, AZ, 85719, USA; 8 Biomedical Research Foundation, Academy of Athens, 4 Soranou Ephessiou, Athens, 11527, Greece; 9 HTW Berlin University of Applied Science, Wilhelminenhofstraße 75A, Berlin, 12459, Germany; 10 BPH INSERM1219 & LaBRI, Univ. Bordeaux, 146 rue Léo Saignat, F-33000, Bordeaux, France; 11 Normandie Univ, UNIROUEN, CHU Rouen, Department of Clinical Research, Rouen University Hospital, 1 Rue de Germont, Rouen Cedex, 76031, France; 12 ZB MED Information Centre for Life Sciences, Gleueler Str 60, Cologne, 50931, Germany; 13 University of Notre Dame Center for Research Computing, 814 Flanner Hall, Notre Dame, IN, 46556, USA; 14 National Institute of Allergy and Infectious Diseases, National Institutes of Health, 5601 Fishers Lane, Rockville, MD, 20852, USA; 15 Institute of Computing, University of Campinas, Av Albert Einstein 1251, Campinas, São Paulo, 13082-853, Brazil; 16 Universidad Carlos III de Madrid, C/ Madrid, 128, Getafe (Madrid), 28903, Spain; 17 250D Navari Center for Digital Scholarship, Hesburgh Library, University of Notre Dame, Notre Dame, IN, 46556, USA; 18 School of Data Science, University of Virginia, P.O. Box 400249, Charlottesville, VA, 22904, USA; 19 Leibniz Institute for Prevention Research and Epidemiology, Achterstrasse 30, Bremen, 28359, Germany; 20 Karolinska Institutet & Swedish National Data Service, Nobels väg 9, Stockholm, 17177, Sweden; 21 Cancer Research UK, 2 Redman Place, London, E20 1JQ, UK; 22 University of Southampton, University Road, Southampton, SO17 1BJ, UK; 23 ICPSR, University of Michigan, P.O. Box 1248, Ann Arbor, MI, 48106-1248, USA; 24 OpenEdition/Department of Library Science, Archives and Information Systems, International Hellenic University, P.O. Box 141, Thessaloniki, 57400, Greece; 25 Institute of Applied Biosciences (INAB), Centre for Research and Technology Hellas (CERTH), Thessaloniki, 57001, Greece; 26 INRAE National Research Institute for Agriculture, Food and Environment, 147 Rue de l'Université, Paris, 75007, France; 27 Laurentian University, Ontario, P3E 2C6, Canada; 28 Oxford e-Research Centre, Department of Engineering Science, University of Oxford, 7 Keble Road, Oxford, OX1 3QG, UK; 29 Department of Computer Science, Royal Holloway, University of London, Bedford Building, Egham, TW20 0EX, UK; 30 Indepedent Researcher, Strada Costiera, Trieste, 34151, Italy; 31 Division of Rheumatology, Orthopedics and Dermatology, School of Medicine, University of Nottingham, Queens Medical Centre, Nottingham, NG7 2UH, UK; 32 Ribeirão Preto Medical School, University of São Paulo, São Paulo, Brazil; 33 Modestum Ltd, Hilton, Derbyshire, UK; 34 Research Data Alliance - US Region (RDA-US), c/o Ronin Institute, 127 Haddon Place, Montclair, NJ, 07043, USA

**Keywords:** Open science, Sharing research outputs in pandemics caused by infectious diseases, FAIR and CARE principles, Omics, Epidemiology, Social Science, Clinical Research, COVID-19

## Abstract

The systemic challenges of the COVID-19 pandemic require cross-disciplinary collaboration in a global and timely fashion. Such collaboration needs open research practices and the sharing of research outputs, such as data and code, thereby facilitating research and research reproducibility and timely collaboration beyond borders. The Research Data Alliance COVID-19 Working Group recently published a set of recommendations and guidelines on data sharing and related best practices for COVID-19 research. These guidelines include recommendations for clinicians, researchers, policy- and decision-makers, funders, publishers, public health experts, disaster preparedness and response experts, infrastructure providers from the perspective of different domains (Clinical Medicine, Omics, Epidemiology, Social Sciences, Community Participation, Indigenous Peoples, Research Software, Legal and Ethical Considerations), and other potential users. These guidelines include recommendations for researchers, policymakers, funders, publishers and infrastructure providers from the perspective of different domains (Clinical Medicine, Omics, Epidemiology, Social Sciences, Community Participation, Indigenous Peoples, Research Software, Legal and Ethical Considerations). Several overarching themes have emerged from this document such as the need to balance the creation of data adherent to FAIR principles (findable, accessible, interoperable and reusable), with the need for quick data release; the use of trustworthy research data repositories; the use of well-annotated data with meaningful metadata; and practices of documenting methods and software. The resulting document marks an unprecedented cross-disciplinary, cross-sectoral, and cross-jurisdictional effort authored by over 160 experts from around the globe. This letter summarises key points of the Recommendations and Guidelines, highlights the relevant findings, shines a spotlight on the process, and suggests how these developments can be leveraged by the wider scientific community.

## Disclaimer

The views expressed in this article are those of the author(s). Publication in Wellcome Open Research does not imply endorsement by Wellcome.

## Introduction

The coronavirus disease 2019 (COVID-19) pandemic is currently one of the most challenging global issues, with economic, social, political, cultural and scientific consequences ( Nicola *et al.*, 2020; Rajkumar, 2020). The rapid spread of the severe acute respiratory syndrome coronavirus 2 (SARS-CoV-2) virus and the need for global stewardship have led researchers to collaborate on a worldwide scale, escalating the production of scientific data and highlighting the urgency to provide those data in an accessible, re-usable, and timely manner.

To ensure the rapid sharing of high-quality data, the Research Data Alliance (RDA) established a rapid-response working group on COVID-19, which quickly grew to more than 600 members, with over 160 individuals contributing actively to recommendations over a 10 week period ( Callaghan, 2020). The working group was divided into four research areas (Clinical, Omics, Epidemiology, Social Sciences) with four cross-cutting themes (Community Participation, Indigenous Data, Research Software, Legal and Ethical Considerations). Despite scheduling challenges across multiple time zones, weekly writing sprints were organised starting with a subgroup discussion, effectively a scrum deciding what issues should be addressed with all subgroups feeding back to one another about progress and specific topics and recommendations. After an overall editorial team had overseen the final editing of all the recommendations, the final draft was released to the wider community for review and comment. Individual working groups were tasked with addressing any issues raised.

The cross-cutting themes were selected as they have impact on all four research areas. Community Participation highlights the work done by communities who are collecting, curating, and sharing data with the goal of improving research outputs and public knowledge. Each of the research areas are dependent on these activities. Indigenous Peoples are acutely impacted by the negative social, economic, environmental and health outcomes of COVID-19 ( UN Special Rapporteur on the rights of Indigenous Peoples, 2020) and hence researchers must be aware of their particular needs. Legal and Ethical Considerations are necessary to inform researchers, practitioners and policymakers on how to deal with these aspects of pandemic response ( European Group on Ethics in Science and New Technologies, 2020; UNESCO, 2020; WHO, 2007), balancing principles of openness with concerns related to human rights and dignity ( Council of Europe, 2020). Research Software is key in conducting research ( Nangia & Katz, 2017). Developing and publishing research software (discussed in the RDA document) enables reproducibility, rapid development, and correction of the software and hence impacts all the above four research areas.

The objective of this RDA working group was to provide data sharing recommendations for researchers, clinicians, policymakers, funders, publishers, and providers of infrastructure concerning the most important challenges encountered during the current pandemic.

The final version of the RDA COVID-19 Recommendations and Guidelines on Data Sharing ( RDA COVID-19 WG, 2020) was released on 30th June 2020 and provides up-to-date advice across the eight areas mentioned above to support robust data sharing and meaningful data reuse for the COVID-19 pandemic management. Each sub-section of the 143-page document is organised into four main subparts: “ *Focus and Description”, “Scope”, “Policy recommendations”* and “ *Guidelines”*, allowing efficient navigation for the reader seeking precise information. In addition, there was the possibility for sub-sections to provide a link to additional supporting output in the form of discussion papers or preprints ( RDA COVID-19 Epidemiology WG, 2020).

Prefaced by an executive summary, the Recommendations and Guidelines provide an essential reference text for the above stakeholders. Each section provides high level recommendations to policymakers and funders, followed by more granular guidelines for the other stakeholders. It is also supported by an extensive curated bibliography publicly accessible online Zotero Library ( RDA COVID-19 WG, 2021) and which continues to be updated to support ongoing work following publication of the Recommendations and Guidelines. An infographic was created to provide an overview and highlight key areas. Two prototype tools are under development: (a) the DS Wizard, that allows readers to pull out content to create an abridged version focused on their specific interests; and, (b) a mind map to assist readers in exploring the document. These prototype tools under development are available on the Value of RDA for COVID-19 webpage. The comprehensive recommendations and guidelines and related navigation tools facilitate uptake by all stakeholders (including the public), who wish to access reliable information on the global COVID-19 research and response process.

Since disciplines and communities often develop *ad hoc* data management practices that are prone to becoming siloed, the report encourages interoperability and data exchange between stakeholders. It highlights the advances and procedures in different disciplines, but crucially also draws attention to commonalities across disciplines, fostering interdisciplinary action, understanding of the disciplines that stakeholders are not part of, and future collaboration. Although recommendations are most frequently aimed at stakeholders such as researchers, they also include items of relevance to policy and decision-makers around governance and data protection, legislation, and funders in encouraging appropriate planning from the outset for managed data sharing. Explicit guidance per stakeholder is also provided in the navigation tools accompanying the recommendations.

The RDA is well positioned to develop such guidance due to its grassroots, participative tradition of interdisciplinary self-motivated dialogue and solutions-based outputs. Indeed, the sheer number of experts globally prepared to commit time during the very tight timeline imposed by the public health emergency makes the recommendations a model example for such all-encompassing collaboration. Beyond definitive guidance, the output may also serves to encourage further discussion and action. It is a significant body of work highlighting a common understanding and motivation to share knowledge across the research community.

## Subgroup recommendations

In this section, we provide a brief motivation for each sub-group, the problems identified, and a summary of key recommendations per group as well as overarching guidance. As mentioned briefly above, there were four discipline-specific subgroups (Clinical to Social Sciences guidelines, below), focusing on the specific challenges of their domain. However, an important feature of the recommendations were the four complementary working groups (Community participation to Legal and Ethics guidelines, below) described as ‘cross-cutting’ because of their relevance across the four discipline-specific subgroups, ultimately they provide a much needed domain agnostic-perspective on the one hand relevant to all groups but also typical of the need during such public emergencies to review and understand global issues which inform and unite different aspects of research.

One common motivator for the recommendations is to find mechanisms that allow data sharing whilst maintaining appropriate governance. In consequence, the Social Science recommendations, for example, emphasise both normative and technical interoperability, as well as harmonized access to curated data repositories, whilst encouraging a suitable balance between the rights of those providing data and the potential benefits to the community. This topic is addressed in detail by the Community participation subgroup especially from the perspectives of app development for community-generated data (for tracking and contact tracing), collaborative data collection and stewardship.

### Clinical guidelines

Healthcare measures and clinical research are at the forefront of combating the COVID-19 pandemic. Obtaining actionable clinical information about the disease and seeking an effective treatment to fight the infection are key to minimising the impact of this unprecedented global health challenge. The focus was on data in clinical trials and clinical care outside clinical trials. Standards on immunological, imaging, and other healthcare data are identified. Clinical trials should follow the International Council for Harmonisation (ICH) efficacy guidelines to ensure the data quality. As cases rise, the promotion of clinical data sharing is of utmost importance. Many studies and trials are performed under enormous time pressure, which can weaken the methodology and lead to preliminary results being published without a full review. We recommend making the data underlying the research available alongside the research results. The recommendations detail how to use trustworthy repositories to provide transparency, integrity, and context to data for timely discovery and the validation of new findings. A key goal is to avoid policymaking based weak or fraudulent studies, which in turn causes distrust in science ( The Editors of the Lancet Group, 2020).

### Omics guidelines

Omics-scale studies of SARS-CoV-2 are emerging rapidly with exceptional potential to unravel the mechanisms of the COVID-19 pathobiology. These studies offer new mechanistic insights into the pathogenesis of COVID-19 and ways forward for diagnostic and therapeutic intervention, while at the same time generating a tremendous amount of data. The Omics subgroup was motivated to draft guidelines based on the requirement for rapid, open data sharing. This rapid sharing facilitates early insights into the molecular biology of the COVID-19 processes at a cellular level, possibly leading to new therapeutic targets, diagnostic markers, and disease management. Omics research should be a collaborative effort to learn the genetic determinants of COVID-19 susceptibility, severity, and outcomes. Thus, the use of domain-specific repositories to enable standardisation of terms and enforce metadata standards is mandated. Availability and re-usability of research data on COVID-19 in order to prevent unnecessary duplication of work is described for virus genomics, host genomics, proteomics, metabolomics, lipidomics, and structural data. The RDA Omics sub-group provides clear recommendations of repositories to find existing data depending on the target methodology in the above research areas, as well as best practices for sharing data and identifying the most prevalent data and metadata formats.

### Epidemiology guidelines

An immediate understanding of the COVID-19 epidemiology is crucial to slowing infections, minimising deaths, making informed decisions about when, and to what extent, to impose mitigation measures, and when and how to reopen society. One of the major challenges encountered in COVID-19 epidemiology is that data and models are not comparable or interoperable, and they are frequently incomplete, provisional, and subject to correction under changing conditions, making their use and reuse for timely epidemiological analysis extremely challenging. The principal guidelines for researchers are to ensure that the data models are inclusive of not only clinical data, disease milestones, indicators and reporting data, but also contact tracing and personal risk factors. Our recommendations for policymakers are to incentivise the publication of situational data, analytical models, scientific findings, and reports used in decision making based on common standards so that they are reusable by epidemiologists. The supporting output ( RDA COVID-19 Epidemiology WG, 2020) expand upon six focus areas (data sources, instruments, privacy, epidemiological data model, computable framework, and an epi-stack framework) to progressively develop a data driven global vision for managing novel biological threats such as COVID-19.

### Social sciences guidelines

The social sciences recommendations seek to ensure that social science data are widely (re)usable to answer fundamental questions about social aspects of the pandemic, and that the data are accessible for work ongoing in other domains. Given the cross-disciplinary and pervasive nature of social science data especially during socially disruptive events such as the coronavirus pandemic, the recommendations focus more specifically on the normative and technical aspects of data interoperability and exchange. The subgroup recommendations therefore include: encouraging data management that follows best practices and improves data sharing; use of trustworthy repositories to share data; retention of information (e.g., geographic information) to allow data linkage within and across domains while maintaining confidentiality; access to measures that are useful when making statistical adjustments for selection bias, thereby improving the representativeness of findings from limited samples; and balancing the desire to share data widely while ensuring the protection of human subjects and that confidential data are kept secure.

### Community participation guidelines

Community participation guidelines were created with the aim of bridging stakeholder involvement and ensuring that inputs from researchers, citizen scientists, developers and device makers are streamlined, while perspectives from patients, policymakers and the public at large are also considered. Linking communities and supporting communication is essential for coordination and avoiding duplication of efforts since many communities are driving similar or complementary efforts in response to the current public health emergency. These recommendations aim to support the varied work of communities in sharing data to improve research outputs and public knowledge and provide a set of guidelines designed to ensure an approach based on best participatory practices. Furthermore, the Community Participation guidelines also aim to enable citizen scientists undertaking research to contribute to a common body of knowledge, and to encourage public and patient involvement (PPI) throughout the data management lifecycle from research question to final data sharing and usage especially in the emergency contexts that require their direct and active engagement.

### Guidelines for data sharing respecting indigenous data sovereignty

Indigenous Peoples and nations globally need to be actively engaged in governance processes that include Indigenous-related COVID-19 data, data lifecycles, and data ecosystems. This is a necessary part of respecting the inherent rights of Indigenous nations to have sovereignty and governance over Indigenous data. The Indigenous COVID-19 data guidelines set out the minimum requirements for Indigenous-designed data approaches regarding governance, collection, ownership, application, sharing, and dissemination of Indigenous data, specifically in relation to COVID-19. These guidelines reflect and support Indigenous Data Sovereignty (see www.GIDA-global.org), underpinned by the United Nations Declaration on the Rights of Indigenous Peoples ( UNDRIP) and framed around the CARE (for Collective Benefit, Authority to Control, Responsibility, Ethics) Principles for Indigenous Data Governance. These guidelines do not supersede or replace existing Indigenous governance protocols or agreements developed (or under development) by Indigenous Peoples or nations. Rather, they point to the need for Indigenous Peoples and nations to be engaged in governance on their own terms across COVID-19 data lifecycles and ecosystems, so they are aligned to ethical and cultural Indigenous data practices supported by collective consent. This demands proactive investment in Indigenous community-controlled data infrastructures to support community capacity and resilience and improvement of the flow of information for effective public health response.

### Software guidelines

Regardless of the research domain, software plays a fundamental role to realise reproducible science as it enables analyses and processing of data. The recommendations for research software cover aspects of development, release and maintenance, derived from previous work ( Akhmerov *et al.*, 2019; Anzt *et al.*, 2020; Clément-Fontaine *et al.*, 2019; Jiménez *et al.*, 2017; Lamprecht *et al.*, 2019; Wilson *et al.*, 2017). Our recommendations to researchers focus on key practices enabling (re)use of research software making it easier for other researchers to build upon and focus their efforts on new approaches. Openness, availability, documentation and examples are key elements here. Before software is re-used, it must be found; therefore, our recommendations focus on software citation, archives and deposit platforms for released versions and alignment with publishing best practices. Finally, neither software development nor its publication are possible without sufficient funding support. In this sense, we centred our recommendations on increasing the recognition of software, its role in reproducibility, and funding opportunities not only for development but also for maintenance and sustainability.

### Legal and ethics guidelines

Data sharing must occur in compliance with relevant legal and ethical frameworks. Especially for health-related data, this frequently leads to tension between the interests of the individual and of society in general. This section therefore makes recommendations to help ensure best practices are respected in using COVID-19 data across jurisdictions and institutions. The guidelines draw attention to approaches which may be of particular interest to policymakers and regulators. These recommendations include a synthesis of foundational principles of data privacy in law and ethics, a description of organisational data governance practices, and sources of legal and ethical obligations applicable to researchers performing studies during COVID-19, including biomedical and social science research ethics guidance. Data governance is considered throughout the data lifecycle in the spirit of community engagement and benefit sharing and leads on to a discussion of the distinct consent standards applicable to clinical care, research ethics, and data privacy law. Practical pointers help researchers identify the most appropriate actor at their institution to guide them in adhering to local legal and ethical requirements, while best practices for data de-identification and anonymisation, as well as data and software IP licensing are described. In many instances, sharing data with external researchers or transferring data to a third country can engage competing legal responsibilities and create legal ambiguities or impose conflicting obligations on researchers. Since multiple regulatory regimes may apply simultaneously to a single instance of data use or data sharing, therefore, the recommendations emphasize the need for research institutions and regulatory bodies to participate in the clarification and interpretation of the ethical and legal principles for data use, data sharing, and international data transfer.

### Overarching recommendations

In addition to each group’s recommendations, the document starts with a series of overarching recommendations. These foundational elements draw directly from the findings of the subgroups, as well as from broader current discussions on research data sharing and Open Science, tailored to the critical need for timely, precise, and technically interoperable research data sharing under a pandemic.

The sharing of research data promotes research integrity, enables others to investigate results, and fosters the very purpose of research itself - to build upon existing knowledge towards new discoveries. The timely sharing of well-curated data (and software, algorithms, and other resources) enables reuse, often for purposes unanticipated by the research that first produced the data. For this reuse to be possible, data must be collected, documented, curated, preserved, and made available through trusted and recognised platforms. The FAIR data principles ( Wilkinson *et al.*, 2016) - promoting data to be Findable, Accessible, Interoperable and Reusable - provide a well-recognised framework for data sharing and were noted frequently by contributors across the sections. Ethical and reproducible data were also emphasized, leading to the the concept of FAIRER data.

Disciplinary borders provide one challenge, but so do geographical and administrative boundaries. COVID-19 does not respect borders of any kind, so, similarly, neither can research. The need for cross-jurisdictional efforts to support sharing of data and other resources, through coordination, funding and legal agreements, is also key. Computational infrastructures need to be refreshed and invested in as a public good; investment in technology needs to be accompanied by support for human resources to maintain infrastructure, and training programmes in data stewardship need to be developed and offered broadly. Data and other outputs need to be prepared for external sharing and secondary use so that they are understandable, and this process should be started as early as possible in the research process with the creation of a data management plan (DMP), which details how data are stewarded throughout the research lifecycle. This lifecycle is key to the remaining ‘Foundational’ elements: data must be accompanied by documentation such as research methods, context, data manipulation; rich metadata in standard formats need to accompany outputs; data should be deposited in domain-suitable trustworthy data repositories for discovery, preservation, and reuse; and, the rapid publication of data should be encouraged supported, and mandated by funders and publishers.

## Discussion

A key aim of the recommendations and guidelines has been to offer both system-wide and concrete guidance to facilitate data sharing among researchers from multiple disciplines and the transfer of data across geographical boundaries in a timely and accurate manner, thus helping accelerate the time to a cure, supporting informed decisions and improving the global response to the pandemic.

The involvement of specialists and practitioners coming from the many disciplines and fields impacted by the pandemic has ensured that the report is both expert-informed and community reviewed. The incorporation of repeated open consultations was also meant to facilitate a fast-track path to wider adoption, considering that researchers, policymakers, and other stakeholders have been involved as early as possible in the formulation and drafting of a consensus document. The priority is to encourage wide adoption of these guidelines and recommendations in order to help accelerate successful solutions to the pandemic.

Instead of a silo-based approach, the document identifies the commonalities in data management across different research areas and themes. It is the result of a standardised common approach in how the different sections were drafted, structured and reviewed. Identifying commonalities implies that similar solutions can be identified and applied. This bridge from the STEM (Science, Technology, Engineering, and Maths) to social science aspects of the COVID-19 challenge demonstrated how truly interdisciplinary work can provide valuable insights and stimulate a creative process. The added value of such overarching collaboration is a key takeaway from this process that may also enrich similar efforts.

The document was developed with a comparatively light level of moderation and emerged on a very rapid timeframe of 10 weeks, including the release of five drafts posted for open feedback and comments on a weekly basis. Writing coordination focused on ensuring the flow of information, so sub-groups, moderators and chairs met regularly. There was a weekly public webinar, as well weekly Co-Chairs meetings, and weekly coordination sessions for Chairs and Moderators. In addition, Subgroups, led by co-moderators, determined their respective meeting frequency and manner of working, ranging from one to three or more meetings per week depending on the group. Subgroups were responsible for reviewing and resolving any comments received on the previous week’s work. Small teams were set up for visualisation of recommendations, and for managing references across all sub-groups. The foundational elements and executive summary were drafted by the editorial team, undergoing successive editing phases, where participants from different groups could comment widely across the whole document. This light-weight structure was enabled through relatively simple tools, namely Google Docs, Zotero and videoconference calls. The final publication is designed as a reference text, where users are likely to selectively read parts of the document relevant to them, so a certain degree of repetition on key advice was retained to address this selective reading.

Going forward, the RDA COVID-19 initiative has demonstrated that there is a global willingness among experts from a range of disciplines to engage with the grand challenges we face as well as to generously offer their time and experience to generate thorough and well-rounded guidance that is attentive to philosophical and pragmatic differences. This experience made clear that to a great extent, the knowledge, expertise, and solutions for working together in the face of global emergencies are already in place, so we need to foster this through continued coordination, harmonisation, and decision making. Engagement within different stakeholder groups has continued, representatives from the European Commission developed a factsheet and informed partner organisations including The Coalition for Epidemic Preparedness Innovations, WHO, Wellcome, and the Gates Foundation.

This work has led to a number of follow-up activities within the RDA, including the creation of a new Community of Practice for Infectious Disease Data. There were four follow-up sessions at the 16th RDA Plenary in November 2020, and another three are planned for the 17th Plenary in April 2021. There have also been many spinoff activities. One article has been published in a peer-reviewed journal ( Rodriguez-Lonebear *et al.*, 2020), one is currently under review ( Carroll *et al.*, 2021, submitted to Frontiers in Medical Sociology), and one has been conditionally accepted ( Pickering *et al.*, in press, submitted to Open Research Europe), in addition to the present paper. There are a further five papers that have been published as preprints ( Austin *et al.*, 2020; Hallinan *et al.*, 2020; Sauermann *et al.*, 2020; Schmidt *et al.*, 2020; and Tonnang *et al.*, 2020), three that are available as discussion papers ( Greenfield *et al.*, 2020a; Greenfield *et al.*, 2020b; Greenfield *et al.*, 2020c), and one that is available as an RDA supporting output ( Harrower & Dillo, 2020). There have been 10 conference presentations and seven webinars. Finally, the results of this work have supported a number of research grant proposals including two that have been funded relating to COVID-19 and Indigenous communities ( NIEHS, 2020).

These spinoff activities demonstrate a clear direct impact of this initiative for both the advancement of research and the leveraging of “expert-informed” and “community reviewed” resources. The RDA is committed to sharing and improving the approach as an example of good practice, offering its structure, processes, and support as a framework for similar efforts.

## Conclusions

The RDA COVID-19 Recommendations and Guidelines on Data Sharing ( RDA COVID-19 WG, 2020) highlights the importance of data sharing and secondary data use in different domains with respect to COVID-19. It provides a range of detailed guidelines aimed at communities with different practices of data management. The guidelines directly target researchers to facilitate best practices and maximise efficiency while also addressing policymakers, funders, publishers, and providers of data infrastructures with a framework for future emergencies. With over 600 members, the group reached a substantial size with diverse knowledge, background, and domain experience.

The present paper has focused on the above document and the WG. An analysis of other related community activities is beyond the scope of this paper. Going forward, the RDA COVID-19 WG is not only focused on the wider communication and adoption of the recommendations and guidelines themselves but also on providing best practices for the process of developing similar reports and outputs in the context of a multidisciplinary, bottom-up and geographically diverse community, to be able to rapidly respond to acute global challenges such as the COVID-19 pandemic.

The RDA is engaging with stakeholders at various levels to build impact and encourage adoption of the guidelines. From a policy perspective, the WG was instigated rapidly in response to a request by the European Commission. The guidelines can be an important resource for the Organisation for Economic Co-operation and Development (OECD), the Bill and Melinda Gates Foundation, Wellcome, the WHO, the Global Research Collaboration for Infectious Diseases Preparedness (GloPID-R), the European and Developing Countries Clinical Trials Partnership (EDCTP) and the Innovative Medicines Initiative (IMI).

The experience of writing the guidelines demonstrates that the creation of a document with contributions from a large, diverse group is possible in a relatively short amount of time. Subgroups can operate in tandem to save time; however, they require editors to move different sections towards completion, and to help create a consistent structure and approach throughout the final document. A framework to steer the subgroups towards a common goal, particularly in terms of the intended audience, is also crucial. This community-driven writing can serve as a template for future world-wide urgent challenges be it the next pandemic, a natural disaster, or indeed the climate crisis. The urgency and unprecedented global and near simultaneous nature of the pandemic likely contributed to participant motivation. The question remains of how similar large scale, multidisciplinary challenges might be addressed when the urgency is not as palpable. Without such urgency, this might attract fewer contributors. Nevertheless, as described here, this still provides a good mechanism for creating key guidelines that reflect a large diverse community. The process of forming the collaboration and developing the guidelines was also studied as an object of social science research.

## Data availability

No data are associated with this article.
