# Supplementary material for: Fostering global data sharing: highlighting the recommendations of the Research Data Alliance COVID-19 working group
Source: Wellcome Open Res. 2021 May 26;5:267. Originally published 2020 Nov 9. [Version 2] doi: 10.12688/wellcomeopenres.16378.2 (PMC7808050; doi:10.12688/wellcomeopenres.16378.2)
Supplement: Peer review history

# Peer review: Reviewer response for version 2

**DOI:** 10.21956/wellcomeopenres.18520.r44088

**Published:** 2021-07-07

**Authors:** Fruzsina Molnar-Gabor

**Affiliations:** 1 Heidelberg Academy of Sciences and Humanities, Heidelberg, Germany

I have assessed the recent revisions and think they are appropriate.

 I am looking forward to reading the announced further publication that will analyse how this community worked together at speed to deliver the final document.

Does the article adequately reference differing views and opinions?

Yes

Are all factual statements correct, and are statements and arguments made adequately supported by citations?

Yes

Is the Open Letter written in accessible language?

Yes

Where applicable, are recommendations and next steps explained clearly for others to follow?

Partly

Is the rationale for the Open Letter provided in sufficient detail?

Yes

Reviewer Expertise:

NA

I confirm that I have read this submission and believe that I have an appropriate level of expertise to confirm that it is of an acceptable scientific standard.

# Peer review: Reviewer response for version 1

**DOI:** 10.21956/wellcomeopenres.18015.r41351

**Published:** 2021-01-13

**Authors:** Maria Luiza Machado Campos

**Affiliations:** 1 Federal University of Rio de Janeiro (UFRJ), Rio de Janeiro, Brazil

The article presents an overview of the results from the COVID-19 Research Data Alliance Working Group, originally delivered in a report addressing the challenges of data sharing during the COVID-19 pandemic. In that report, the working group discussed a series of recommendations and guidelines divided into four research areas and four cross-cutting themes. The article highlights the key points and main findings associated to these guidelines, also describing their development process.

 As the original WG report is a long and comprehensive document, the article is an important and useful reading. It is not meant to replace the original report, however it gives a general view of the whole content, serving to increase its dissemination in the scientific community and to give a more tangible understanding to those who do not belong to the specific addressed domains or even for those who need to engage in similar initiatives.

 The introduction is very motivating, presenting the working group and the process of designing guidelines and recommendations. It gives an idea of the effort made during development, especially if we consider the number of participants and the short time on which the report was produced. It also includes links to an overview infographic and to other nice associated resources complementary to the report and to the article.

 At the recommendations section, the authors refer to each of the domain and crosscutting themes with their specific requirements and assumptions and complement them with common issues. Perhaps this section could benefit from better balancing the content of the different subgroups or areas. Some are very well described, with a good presentation of results (such as Software guidelines, Omics guidelines), while others, in contrast, are simpler (such as Clinical Guidelines and Community participation guidelines). Community participation, for example, could have its relevance and characterization better justified and described, so that the readers could better understand its priority over other candidate themes.

 During the discussion session, it would be interesting to have some comparison or comments in relation to other existing works addressing directions for the COVID-19 research projects or more general data strategies, as many of these topics have been highly debated in the academic and government areas. Maybe the joint effort Data Together, involving RDA, CODATA, GO FAIR and EOSC could have been mentioned. Although the FAIR principles have been cited, initiatives like GO FAIR VODAN IN (the Virus Outbreak Data Network) were not discussed. Such works could certainly complement the RDA WG results and serve as further references for the readers.

 Obs: The article is well presented and written, but please correct “The recommendations for research software COVER aspects…” (and not COVERS).

Does the article adequately reference differing views and opinions?

Partly

Are all factual statements correct, and are statements and arguments made adequately supported by citations?

Yes

Is the Open Letter written in accessible language?

Yes

Where applicable, are recommendations and next steps explained clearly for others to follow?

Partly

Is the rationale for the Open Letter provided in sufficient detail?

Yes

Reviewer Expertise:

Data Management, Information integration, Ontology engineering.

I confirm that I have read this submission and believe that I have an appropriate level of expertise to confirm that it is of an acceptable scientific standard.

# Response

**Published:** 2021-04-02

**Authors:** Hugh Shanahan

**Affiliations:** Royal Holloway, University of London, UK

We thank the referee for her comments and have addressed the points made. We list below the comments made and our response to each.

*“Recommendations section: could benefit from better balancing the content of the different subgroups or areas. Simpler section such as Clinical Guidelines and Community participation guidelines. Community participation, for example, could have its relevance and characterization better justified and described, so that the readers could better understand its priority over other candidate themes.“*

**The relevance of the different cross-cutting themes, including Community participation,  has been explained in more detail in section 1, paragraph 3. Furthermore the Community Participation overview has been additionally clarified in section 2.5. The section on clinical guidelines has also been updated.**

*“Discussion section: It would be interesting to have some comparison or comments in relation to other existing works addressing directions for the COVID-19 research projects or more general data strategies. Maybe the joint effort Data Together, involving RDA, CODATA, GO FAIR and EOSC could have been mentioned.”*

**In section 6 (discussion) paragraph 5 the impact of other initiatives is discussed.**

*“Obs: The article is well presented and written, but please correct “The recommendations for research software COVER aspects…” (and not COVERS).”*

**This error has been corrected.**

# Peer review: Reviewer response for version 1

**DOI:** 10.21956/wellcomeopenres.18015.r41353

**Published:** 2021-01-07

**Authors:** Fruzsina Molnar-Gabor

**Affiliations:** 1 Heidelberg Academy of Sciences and Humanities, Heidelberg, Germany

The article is a timely roadmap for communally and consistently dealing with the crucial global challenges arising from the COVID-19 pandemic (as they pertain to data sharing). It delivers substantive cross- and inter-disciplinary guidance and recommendations for those stakeholders able to influence the international response to these challenges. That the RDA was able to deliver such a comprehensive report within the extremely limited timeframe of ten weeks is down to just how well-situated the Alliance is to take up the function of organizing and establishing interdisciplinary responses to global challenges and how well-placed it is – through its organizational and management efforts and its access to and use of relevant expertise – to deliver substantial answers to thorny questions arising from the current crises. On the other hand, even the convenient situation and placement of the RDA would not have been enough to respond adequately without its determined work early in the pandemic to establish a working group on COVID-19, which was crucial to shaping the RDA’s response.

 Indeed it is this very procedure – of developing an adequate response in the form of recommendations and guidelines to foster data sharing – that is most relevant for both the current pandemic and any potential future global challenges of comparable nature, as these require solutions which transcend individual communities and disciplines and can only be reached by joining forces. While respecting and appreciating the knowledge and professional expertise of all members that have contributed to this remarkable result, it is only the final presentation of the results of the report in the article and not the substantive content of the report itself that can be subject to this present review.

 Thus, this review is motivated by the assumption that readers will most probably read the article before engaging with the whole report. Accordingly, this review aims to ensure the article provides a clear representation of the results. Additionally, while the report has an Executive Summary which should not be replaced by the article, the article would be a good place to elaborate on the implementation and application of the guidance found within the report.

 To begin the review, I would first like to address the structure and main terms of the article as they relate to the structure of the report:

 Firstly, the article presents a good summary of the results of the working group in a manner likely to engage readers and encourage them to read the main report, as it points out structural-substantive elements that could draw readers’ interest.

 While the presentation of the structural parts is well-balanced, a more detailed presentation of the four cross-cutting themes would be appreciated, as these are of interest for all four research areas. A more detailed summary would benefit readers of all disciplines, interests and purposes.

 Secondly, there are minor inconsistencies in the structure of the document that should be remedied. For example, the title of the ‘Recommendations’ section is slightly confusing given the section relates to both recommendations for policymakers and funders and guidelines for researchers and clinicians. Additionally, the name of the ‘Overarching Recommendations’ subsection is confusing due to the overlap of the terms used, not just in terms of vocabulary but also metatextually in how it alters the presentation of the results. Adding the specific level of recommendations (such as foundational-overarching, area-specific or both) to the subtitles in the relevant subsections would help readers quickly assess the structure.

 Thirdly, there is no substructure to help differentiate between the guidance in each of the four research areas and those relating to the overarching themes, so the ‘Recommendations’ section could also be adapted to mirror the main structure of the report here.

 The second set of suggestions relates to the usage of certain terms that play a substantive role in the article.

 Firstly, the ‘Introduction’ section and the abstract name different addressees (clinicians are missing in the abstract). It is always valuable to include the full list of addressees wherever possible, particularly in the abstract, not just to provide readers with complete information but also to attract relevant stakeholders.

 Secondly, “data sharing”, “data reuse” and “data exchange” seem to be used in the text as equivalent terms but in many jurisdictions – and when examined ethically – they are not entirely interchangeable. This is noted less to spark an academic discussion about interpretative approaches to these notions or about partially-missing binding legal definitions, but more due to the fact that this vocabulary is central to the content as a whole and should, for the purposes of this article, be further harmonized.

 Thirdly, “data managers” seems to be being used as an overarching term for selected addressees. Whether this is in fact the case and how the term and the group of actors included relate to the other, explicitly highlighted groups of addressees needs to be clarified.

 Fourthly, technical and legal/normative interoperability should be – where relevant – labelled as such.

 The third set of suggestions relates to the presentation of the guidance.

 Firstly, in the area of social sciences, the guidelines and recommendations are more strongly connected than in other areas due to the characteristics of this field. This could be emphasized in the article, as is also recommended by the detailed presentation in the report.

 Secondly, in the section ‘Legal and ethics guidelines’, as well as elsewhere in the article, legal challenges could be better emphasized in general to motivate legislators and relevant policymakers to push for frameworks for open infrastructures and rules for the legal securing of data sharing. This is especially relevant in a situation where weighing contradictory legal and ethical positions – usually so difficult in the traditional setting of health data sharing – is already gradually becoming easier and where the pandemic is now forcing a remarkable shift towards an overlap between originally contradictory poles. Emphasizing addressees’ interests is also crucial for grounding their legal positions and the weighing of interests will always ultimately also be guided by those of the public and society (similarly indicated in the report itself, cf. Executive Summary, subsection ‘Recommendations’, p. 9; Section 6. Data Sharing in Social Sciences, subsection 6.4.4, p. 47; Section 10. Legal and Ethical Considerations, subsection 10.2, p. 67).

 Thirdly, the section on legal and ethical guidelines could be more clearly structured to summarize and separate recommendations for policy makers and other related addressees (such as providing the conditions for the relevant actors to be able to work according to the FAIR principles), and guidelines for researchers and other related addressees. This seems particularly important as the report itself emphasizes the role of law (as related to open science through policy: Executive Summary, subsection ‘Recommendations’, p. 8; related to the implementation of legal frameworks that promote sharing of data across jurisdictions and sectors: 10. Legal and Ethical Considerations, subsection 10.3.1, Nr. 9, p. 9).

 The fourth set of suggestions concerns the ‘Discussion’ and ‘Conclusions’ sections.

 The authors consistently and correctly use “best practices” in the plural, clearly recognizing that the developed recommendations and guidelines are also the quintessence of best practices for data sharing. Given this, the ‘Discussion’ section would benefit from placing more emphasis on the standardizing work done on the subject matter of the report. This could be achieved by at the very least elaborating on the direct and indirect effects of the work and the delivered results as a condition of the efficient application of best practices.

 As already highlighted, the focus on the procedure of creating the recommendations and guidelines is of the utmost relevance both for responding to the current pandemic as well as in the sense of creating a living document as a blueprint for dealing with future global challenges. Accordingly, the description of the consultation process is incredibly important (“expert-informed”, “community reviewed”). It would therefore be useful to provide more details on the consultation participants, the exact frequency, methods and modi of consultation and how the results of the consultation have been taken into account.

 Secondly, the report can be characterized – suitably – as an open and responsive document. Nevertheless, in the text of the article, it is not yet clearly described whether there might still be a chance for further consultation. If such a chance still exists, which would be understandable given the fast-paced development of the areas focused on in the report since its publication as well as the valuable guidance provided by other scientific communities in the last months, the proposed methods of openness of the report itself should be shortly elaborated upon. Sounding out approaches to developing a “learning” and “living” document would be highly appreciated by various affected communities, even where this requires additional effort from those involved in its creation.

 Thirdly, consultation with additional international organizations such as sub-organizations of the UN (UNESCO, WHO) would be beneficial, should this not yet have occurred, as both sub-organizations of the UN are frequently cited in the report. If consultation has already been conducted, these organizations should be mentioned in the article (besides the OECD).

 The fifth set of suggestions relates to the additional tools cited in the review.

 It would be beneficial to clarify in the article for whom the decision-making tool is intended. All addressees need to make decisions in their respective contexts and impact areas. The tool would thus be perfect for demonstrating the implementation and application of the recommendations and guidelines through use-case models, and I highly recommend its use as such. It would allow the various addressees to see for themselves the applicability and helpfulness of the report’s guidance in real-life scenarios.

 Furthermore, there is the question of the relationship between the decision-making tool and the DS Wizard Navigation Tool – are these the same, and, if not, what is the connection between them? (Unfortunately, I could not register to try the tool.)

 The question also arises as whom the mind map is intended for. Clarification in the article would be helpful as to whether it relates to a specific circle of addressees or instead presents the proceeding of the working group / drafters / contributors. Furthermore, although this is a technical issue, the mind map does not fit on a standard laptop monitor. While it obviously still needs to be captured at a glance, the textual descriptions can be blended in as notes and zoom and filter functions do exist, an additional, more structured version made for reading on smaller devices would be useful.

 The infographic is an important tool for communicating the results. While it is appreciated that it presents the essence of the results in an easily understandable and consumable fashion, some minor extensions by just one or two words in the relevant places could better emphasize the main results in relation to the relevant parts of the report presentation. Although not explicitly relevant for the text of the article, the weighing of “ethics vs privacy” is slightly generalized in the infographic. A more nuanced view on ethics might be transmitted with one or two additional adjectives, which would also better communicate the balanced results of the report.

 Finally, and in summary, I would like to emphasize – in addition to the breadth of interdisciplinary effort that makes the initiative and the report stand out among other endeavors – two crucial aspects of the relevance of the report that could be better highlighted in the article. First the report underlines “[t]he priority […] for these guidelines and recommendations”, i.e., “to be widely adopted in order to accelerate solutions to the pandemic”. This wide adoption will be achieved by the application of the guidelines and recommendations, with said application creating their inherent consequence – rules of conduct further crystallizing best practices (cf. comments on the decision-making tool above).

 Secondly, the importance of the process of developing the report could be further highlighted, as the process includes the involvement of and consultation with stakeholders and the implementation of their approaches throughout the development procedure of the guidelines and recommendations. The development procedure can – through suitable deliberation – inherently foster the substantive appropriateness of the content. The presentation of the report in the article should justifiably demonstrate this interconnectedness.

Does the article adequately reference differing views and opinions?

Yes

Are all factual statements correct, and are statements and arguments made adequately supported by citations?

Yes

Is the Open Letter written in accessible language?

Yes

Where applicable, are recommendations and next steps explained clearly for others to follow?

Partly

Is the rationale for the Open Letter provided in sufficient detail?

Yes

Reviewer Expertise:

Law.

I confirm that I have read this submission and believe that I have an appropriate level of expertise to confirm that it is of an acceptable scientific standard, however I have significant reservations, as outlined above.

# Response

**Published:** 2021-04-02

**Authors:** Hugh Shanahan

**Affiliations:** Royal Holloway, University of London, UK

We thank the referee for her comments and have addressed the points made. We list below the comments made and our response to each. 

*“While the presentation of the structural parts is well-balanced, a more detailed presentation of the four cross-cutting themes would be appreciated, as these are of interest for all four research areas. A more detailed summary would benefit readers of all disciplines, interests and purposes.“*

**A more detailed summary and presentation of the four cross-cutting themes have been made (section 1, paragraph 3) and an explanation why it is relevant in an emergency situation is explained at the start of section 2.**

*”Secondly, there are minor inconsistencies in the structure of the document that should be remedied. For example, the title of the ‘Recommendations’ section is slightly confusing given the section relates to both recommendations for policymakers and funders and guidelines for researchers and clinicians. Additionally, the name of the ‘Overarching Recommendations’ subsection is confusing due to the overlap of the terms used, not just in terms of vocabulary but also metatextually in how it alters the presentation of the results. Adding the specific level of recommendations (such as foundational-overarching, area-specific or both) to the subtitles in the relevant subsections would help readers quickly assess the structure.”*

**The inconsistencies in the structure of the document have been remedied -  in particular "Subgroup Recommendations" and "Overarching Recommendations" have been separated into two distinct sections.**

*“Thirdly, there is no substructure to help differentiate between the guidance in each of the four research areas and those relating to the overarching themes, so the ‘Recommendations’ section could also be adapted to mirror the main structure of the report here.”*

**These delineations are more concerned with the provenance of the recommendations than the specific audience to which they are directed. Mirroring the structure of the subgroups in the overarching themes section and in the subsections may prove impracticable, as the guidance is drafted in a holistic fashion so as to increase its multidisciplinary appeal.  With this in mind, we have not updated the paper with respect to this.**

*“Firstly, the ‘Introduction’ section and the abstract name different addresses (clinicians are missing in the abstract). It is always valuable to include the full list of addresses wherever possible, particularly in the abstract, not just to provide readers with complete information but also to attract relevant stakeholders.”*

**This inconsistency has been addressed with clinicians added in the abstract.**

*“Secondly, “data sharing”, “data reuse” and “data exchange” seem to be used in the text as equivalent terms but in many jurisdictions – and when examined ethically – they are not entirely interchangeable. This is noted less to spark an academic discussion about interpretative approaches to these notions or about partially-missing binding legal definitions, but more due to the fact that this vocabulary is central to the content as a whole and should, for the purposes of this article, be further harmonized.”*

**Those terms have been harmonized in section 2.8 (Legal and Ethics Guidelines). Specifically the following nomenclature has been adopted: Data sharing is retained as the generic term used to refer to the exchange of data among different groups. The term data reuse is used to connote circumstances in which the secondary use of data raises special technical, operational, or ethical considerations. The term international data transfer is used to refer to instances in which the sharing of data across national boundaries raises particular legal issues. In this section data use, data sharing, and international data transfer are differentiated.**

*“Thirdly, “data managers” seems to be being used as an overarching term for selected addressees. Whether this is in fact the case and how the term and the group of actors included relate to the other, explicitly highlighted groups of addressees needs to be clarified.”*

**The use of such terms has been rationalised in the text, with reference to them removed in section 1, paragraph 6 and and section 2.6.**

*“Fourthly, technical and legal/normative interoperability should be – where relevant – labelled as such.”*

**Technical and legal/normative interoperability have been labelled as such. (Section 2 paragraph 2; section 2.4)**

*“Firstly, in the area of social sciences, the guidelines and recommendations are more strongly connected than in other areas due to the characteristics of this field. This could be emphasized in the article, as is also recommended by the detailed presentation in the report.”*

**The guidelines and recommendations in the area of social sciences have been emphasized (Section 2.4).**

*“Secondly, in the section ‘Legal and ethics guidelines’, as well as elsewhere in the article, legal challenges could be better emphasized in general to motivate legislators and relevant policymakers to push for frameworks for open infrastructures and rules for the legal securing of data sharing. This is especially relevant in a situation where weighing contradictory legal and ethical positions – usually so difficult in the traditional setting of health data sharing – is already gradually becoming easier and where the pandemic is now forcing a remarkable shift towards an overlap between originally contradictory poles. Emphasizing addressees’ interests is also crucial for grounding their legal positions and the weighing of interests will always ultimately also be guided by those of the public and society (similarly indicated in the report itself, cf. Executive Summary, subsection ‘Recommendations’, p. 9; Section 6. Data Sharing in Social Sciences, subsection 6.4.4, p. 47; Section 10. Legal and Ethical Considerations, subsection 10.2, p. 67)”*

**The legal challenges have been better emphasized - specifically a sentence has been added to the ethical and legal section highlighting the instrumental role of research institutions and of regulatory bodies in helping researchers navigate overlapping ethical and legal regimes (end of section 2.8).**

*“Thirdly, the section on legal and ethical guidelines could be more clearly structured to summarize and separate recommendations for policy makers and other related addressees (such as providing the conditions for the relevant actors to be able to work according to the FAIR principles), and guidelines for researchers and other related addressees. This seems particularly important as the report itself emphasizes the role of law (as related to open science through policy: Executive Summary, subsection ‘Recommendations’, p. 8; related to the implementation of legal frameworks that promote sharing of data across jurisdictions and sectors: 10. Legal and Ethical Considerations, subsection 10.3.1, Nr. 9, p. 9).”*

**The section on Legal and ethical guidelines have been reworked such that different sentences are used to highlight the distinct elements of the guidelines directed at the scientific community, and those addressed to policymakers. To ensure that the section adopts the same structure as those of the other subgroups, the section has not been divided into separate paragraphs.**

*“The authors consistently and correctly use “best practices” in the plural, clearly recognizing that the developed recommendations and guidelines are also the quintessence of best practices for data sharing. Given this, the ‘Discussion’ section would benefit from placing more emphasis on the standardizing work done on the subject matter of the report. This could be achieved by at the very least elaborating on the direct and indirect effects of the work and the delivered results as a condition of the efficient application of best practices.”*

**A new paragraph (section 4 paragraph 6)  has been added to address follow up and spin off activities based on the initial work. The discussion section have been emphasized on the standardizing work done elaborating on the direct and indirect effects of the work and the delivered results (section 5, paragraphs 3, 5 and 6)**

*“As already highlighted, the focus on the procedure of creating the recommendations and guidelines is of the utmost relevance both for responding to the current pandemic as well as in the sense of creating a living document as a blueprint for dealing with future global challenges. Accordingly, the description of the consultation process is incredibly important (“expert-informed”, “community reviewed”). It would therefore be useful to provide more details on the consultation participants, the exact frequency, methods and modi of consultation and how the results of the consultation have been taken into account.”*

**The description of the consultation process has been detailed in section 1, paragraph 2 and section 5 (discussion) paragraph 4.**

*“Secondly, the report can be characterized – suitably – as an open and responsive document. Nevertheless, in the text of the article, it is not yet clearly described whether there might still be a chance for further consultation. If such a chance still exists, which would be understandable given the fast-paced development of the areas focused on in the report since its publication as well as the valuable guidance provided by other scientific communities in the last months, the proposed methods of openness of the report itself should be shortly elaborated upon. Sounding out approaches to developing a “learning” and “living” document would be highly appreciated by various affected communities, even where this requires additional effort from those involved in its creation.”*

**As noted above a new section has been added before the Discussion section on further activities based on the initial work with additional references across the document to highlight uptake e.g. section 5, paragraph 6.**

*“Thirdly, consultation with additional international organizations such as sub-organizations of the UN (UNESCO, WHO) would be beneficial, should this not yet have occurred, as both sub-organizations of the UN are frequently cited in the report. If consultation has already been conducted, these organizations should be mentioned in the article (besides the OECD).”*

**We have mentioned consultation with additional international organizations in section 1, paragraph 7 and section 5 (discussion) paragraph 4.**

 “ *It would be beneficial to clarify in the article for whom the decision-making tool is intended. All addresses need to make decisions in their respective contexts and impact areas. The tool would thus be perfect for demonstrating the implementation and application of the recommendations and guidelines through use-case models, and I highly recommend its use as such. It would allow the various addresses to see for themselves the applicability and helpfulness of the report’s guidance in real-life scenarios.*

*Furthermore, there is the question of the relationship between the decision-making tool and the DS Wizard Navigation Tool – are these the same, and, if not, what is the connection between them? (Unfortunately, I could not register to try the tool.)”*

**We have clarified the status of the tools being developed in section 1, paragraph 6.**

*“The question also arises as whom the mind map is intended for. Clarification in the article would be helpful as to whether it relates to a specific circle of addressees or instead presents the proceeding of the working group / drafters / contributors. Furthermore, although this is a technical issue, the mind map does not fit on a standard laptop monitor. While it obviously still needs to be captured at a glance, the textual descriptions can be blended in as notes and zoom and filter functions do exist, an additional, more structured version made for reading on smaller devices would be useful.”*

**We have clarified the role of the mindmap (section 1, paragraph 6). We acknowledge that the mindmap is not optimized for reading on laptops and smaller devices. We also note the text specifies that the mindmap is still in development.**

*“The infographic is an important tool for communicating the results. While it is appreciated that it presents the essence of the results in an easily understandable and consumable fashion, some minor extensions by just one or two words in the relevant places could better emphasize the main results in relation to the relevant parts of the report presentation. Although not explicitly relevant for the text of the article, the weighing of “ethics vs privacy” is slightly generalized in the infographic. A more nuanced view on ethics might be transmitted with one or two additional adjectives, which would also better communicate the balanced results of the report.”*

**The infographic is being updated as suggested.**

*“Finally, and in summary, I would like to emphasize – in addition to the breadth of interdisciplinary effort that makes the initiative and the report stand out among other endeavors – two crucial aspects of the relevance of the report that could be better highlighted in the article. First the report underlines “[t]he priority […] for these guidelines and recommendations”, i.e., “to be widely adopted in order to accelerate solutions to the pandemic”. This wide adoption will be achieved by the application of the guidelines and recommendations, with said application creating their inherent consequence – rules of conduct further crystallizing best practices (cf. comments on the decision-making tool above).”*

**Crucial aspects of the relevance of the report have been emphasized in section 1, paragraphs 7 and 8.**

*“Secondly, the importance of the process of developing the report could be further highlighted, as the process includes the involvement of and consultation with stakeholders and the implementation of their approaches throughout the development procedure of the guidelines and recommendations. The development procedure can – through suitable deliberation – inherently foster the substantive appropriateness of the content. The presentation of the report in the article should justifiably demonstrate this interconnectedness.”*

**This will be discussed at length in a companion another paper that will analyse how this community worked together at speed to deliver the final document. Hence we believe it appropriate to not preempt the findings of that paper.**

# Peer review: Reviewer response for version 1

**DOI:** 10.21956/wellcomeopenres.18015.r41349

**Published:** 2020-11-20

**Authors:** Elena Simperl

**Affiliations:** 1 King’s College London, London, UK

The open letter summarises the results of an RDA working group on COVID-19, which aimed to support worldwide data sharing through interdisciplinary recommendations and guidance. The letter describes the composition of the working group and the process followed to come up with the recommendations. It also lists the main findings for each of the four disciplines of concern as well as some cross-cutting themes such as community participation, law and ethics, and software.

 This is a timely initiative, covering a huge, interdisciplinary space. The recommendations discussed in the letter are aligned with established best practices and knowledge in research data sharing. My only concern is that they remain very generic - speaking as a computer scientist with a reasonable knowledge of data sharing, FAIR, open science, citizen science etc, I found it difficult to:

1. Appreciate what is new about the recommendations compared to the previous excellent work done by the science community in this space. Many of the recommendations have been discussed extensively before in other contexts. It would have been useful to discuss what is truly new and different - there is an argument around the scale and pace of data sharing, but it does not cut through the article.
2. As the guidance remains rather generic, it wasn't straightforward for me to understand how it would be used in practice and by whom. The letter mentions a diverse range of stakeholders, which are no doubt relevant in this context, but the next steps following the guidance are not easy to grasp. The paper should improve on this aspect to ensure the substantial amount of work that the working group has completed bears fruit in the real world.

Does the article adequately reference differing views and opinions?

Yes

Are all factual statements correct, and are statements and arguments made adequately supported by citations?

Yes

Is the Open Letter written in accessible language?

Yes

Where applicable, are recommendations and next steps explained clearly for others to follow?

Partly

Is the rationale for the Open Letter provided in sufficient detail?

Yes

Reviewer Expertise:

Computer science.

I confirm that I have read this submission and believe that I have an appropriate level of expertise to confirm that it is of an acceptable scientific standard, however I have significant reservations, as outlined above.

# Response

**Published:** 2021-04-02

**Authors:** Hugh Shanahan

**Affiliations:** Royal Holloway, University of London, UK

We thank the referee for her comments and have addressed the points made. We list below the changes made to the text to address the comments.

*“My only concern is that they remain very generic - speaking as a computer scientist with a reasonable knowledge of data sharing, FAIR, open science, citizen science etc, I found it difficult to:*

*-          Appreciate what is new about the recommendations compared to the previous excellent work done by the science community in this space. Many of the recommendations have been discussed extensively before in other contexts. It would have been useful to discuss what is truly new and different - there is an argument around the scale and pace of data sharing, but it does not cut through the article.”*

**We now discuss what is truly new and different compared with the work done by the science community in this space (section 1, paragraphs 7 and 8). Aspects related to the exceptional scale and need from timely response have been additionally addressed (section 2, paragraph 1 and 2; section 6 paragraph 5).**

*“ As the guidance remains rather generic, it wasn't straightforward for me to understand how it would be used in practice and by whom. The letter mentions a diverse range of stakeholders, which are no doubt relevant in this context, but the next steps following the guidance are not easy to grasp. The paper should improve on this aspect to ensure the substantial amount of work that the working group has completed bears fruit in the real world.”*

**We have added information on the best way to use the guidance in the real world (section 1, paragraphs 6, 7 and 8).**
